# Prevalence of Incidentally Detected Vascular Compressions in Abdominal Computed Tomography

**DOI:** 10.4314/ejhs.v32i1.8S

**Published:** 2022-10

**Authors:** Mulgeta Getu Kassa, Ferehiwot Bekele Getaneh

**Affiliations:** 1 Department of Radiology, Ambo University Referral Hospital, Ambo, Ethiopia; 2 Addis Ababa University, School of Medicine, College of Health Science, Department of Radiology

**Keywords:** Computed Tomography, median arcuate ligament compression, nutcracker phenomenon, superior mesenteric artery compression

## Abstract

**Background:**

Abdominopelvic vascular structures are exposed to be compressed by adjacent organs or might cause compression of the adjacent hollow viscera. Most of these conditions are asymptomatic and they are detected on imaging incidentally. However, when they are symptomatic, they can lead to a variety of uncommon syndromes in the abdomen and pelvis. Aim of the study was to assess the prevalence of incidental abdominopelvic vascular compressions on computed tomography.

**Method:**

A retrospective cross-sectional study was conducted. All the CT was performed using 64 slice machine. All computed tomography scan of the abdomen between January and April 2019 were evaluated. Data were collected by evaluating abdominal Computed Tomographic scans from Picture archiving and communication system (PACS). Statistical analysis was performed by using SPSS version 25.0 software.

**Results:**

Out of 623 multi detector abdominopelvic computed tomography (MDCT) performed between January 2019 and April 2019; a total of 513 (N = 513) patients were included in the study. This study group comprised of 277 (54 %) females and 236 male (46%) patients. Mean age was 38 ± 20 (mean ± SD). We identified 35(6.8%) participants with imaging features of Superior mesenteric artery (SMA) compressions and a 34(6.6%) with imaging features of nutcracker phenomenon. The celiac artery was compressed by median arcuate ligament (MAL) in 22(4.3%) of them.

**Conclusion:**

Incidentally detected intraabdominal vascular compressions are common to asymptomatic patients. This result emphasizes that, vascular compression syndromes diagnosis should not be made on imaging alone.

## Introduction

Vascular structures in the abdomen or pelvis are liable to be compressed by structures in their vicinity or may cause a compression to the hollow viscera organs(1). The clinical presentations of individuals with these abnormalities may vary from asymptomatic to a significant degree of morbidity. Vascular compression syndromes like median arcuate ligament syndrome, nutcracker syndrome, May-Thurner syndrome, superior mesenteric artery syndrome, ureteropelvic junction obstruction, ureteral vascular compression syndromes and portal biliopathy have characteristic clinical symptoms and a predisposing anatomic variant of vascular structures in imaging. Therefore, diagnosis should be considered carefully in patients with incidentally detected abnormalities in imaging (2).

Nevertheless, imaging plays a role in evaluating the vascular structures and the hemodynamic changes during compression and the adjacent anatomic structures. In addition, imaging directs the surgical approach for patients who are subject to intervention for this condition or other causes.

Doppler ultrasound can be used as a first line imaging, although it is highly an operator dependent and technically difficult to perform it in obese individuals (3).

Barium examination can also be utilized as one of the diagnostic modalities in patients presented with bowel obstruction symptoms and SMA syndrome (duodenal obstruction) (4).

In particular, Multidetector CT (MDCT) has a superior advantage over the others due to its capability of in producing reconstructed images, contrast resolution and less invasive nature. The main drawback of CT is its use of ionizing radiation(5).

MRI is an alternative diagnostic modality when radiation exposure is a high concern and in iodinated contrast allergic patients (6).

**Celiac axis compression**: Harjola et al. first described the compression of the celiac axis by the median arcuate ligament (MAL)(7). The existence of the syndrome and its mechanism of causing pain have been controversial issue since this entity is reported (8).

Diagnosis can be made in CT angiography when stenosis is detected on the axial images, and the celiac artery has a hook or J appearance (9).

**Compression of the duodenum by superior mesenteric artery (SMA)**: The third part of the duodenum can be disposed to compression by superior mesenteric artery due to anatomical or mechanical factors and reduction of the retroperitoneal fat. Various debilitating conditions which cause marked weight loss such as, anorexia nervosa, malabsorption, or hypercatabolic states such as burns, major surgery, severe injuries or malignancies cause retroperitoneal fat loss and acute angulation of SMA (10).

The normal aortomesenteric angle (AMA) and aortomesenteric distance (AMD) ranges 28°–65° and 10–34 mm, respectively (11, 12).

**Left renal vein compression by SMA (Nutcracker phenomenon)**: This phenomenon is characterized by the compression of the left renal vein (LRV) between the SMA and aorta (anterior nutcracker) or the aorta and vertebral body (posterior nutcracker) (1).

The reasons of nutcracker syndrome has been suggested to be posterior renal ptosis, abnormally high course of the LRV and an abnormal SMA branching from the aorta (13).

Abrupt narrowing of the LRV with a triangular shape at the aortomesenteric portion, the “beak sign”, and LRV diameter ratio (hilaraortomesenteric) of ≥4.9 are most sensitive and specific CT parameters to diagnose nutcracker syndrome(14).

The aim of this study is to assess the magnitude of incidentally detected vascular compressions in asymptomatic individuals. Imaging, particularly CT allows evaluation of the anatomic position of these structures and predisposing anatomic and non-anatomical factors. Diagnosis relies on both clinical and imaging findings. However, increasing the awareness of vascular compressions among Radiologists would be important as the disease entity has intermittent character and positive imaging findings should be documented in patient's medical history.

## Materials and Methods

**Figure 1:**
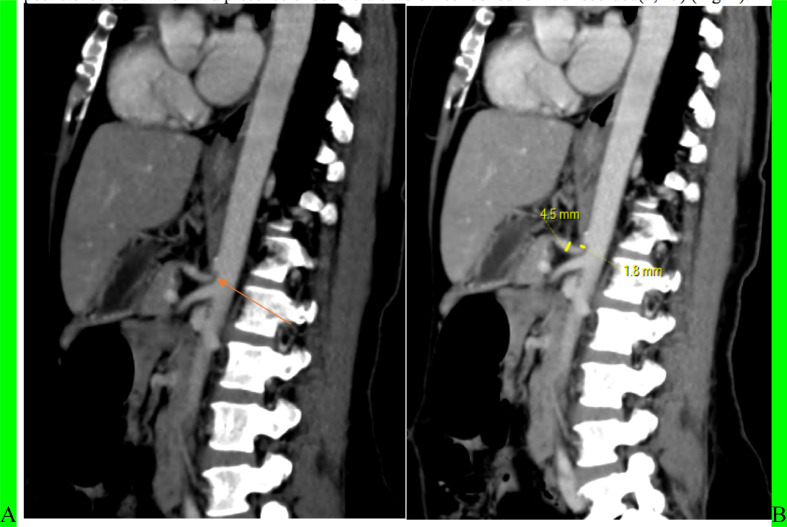
Abdominopelvic CT of 60-year-old, known cervical ca patient. This mid sagittal image shows the celiac artery is compressed by median arcuate ligament (arrow) (A) and has hooked appearance and narrowing (B).

**Image analysis**: A retrospective analysis of 623 MDCT of the abdomen, in whom 64-detector CT was taken for various indications, between January and April was performed. Patients with large abdominal mass which caused distortion of vascular anatomy, poor vascular enhancement and those with suboptimal sagittal reconstruction were excluded from the study. MAL compression of celiac artery was defined by 50% luminal narrowing and hooked appearance on sagittal image with or without presence of ancillary findings. In image with this finding, the vertebral level at which the MAL crossed the celiac axis origin, post-stenotic dilatation and presence of collaterals were also looked for and recorded(9, 15) (Fig. 1).

**Figure 2:**
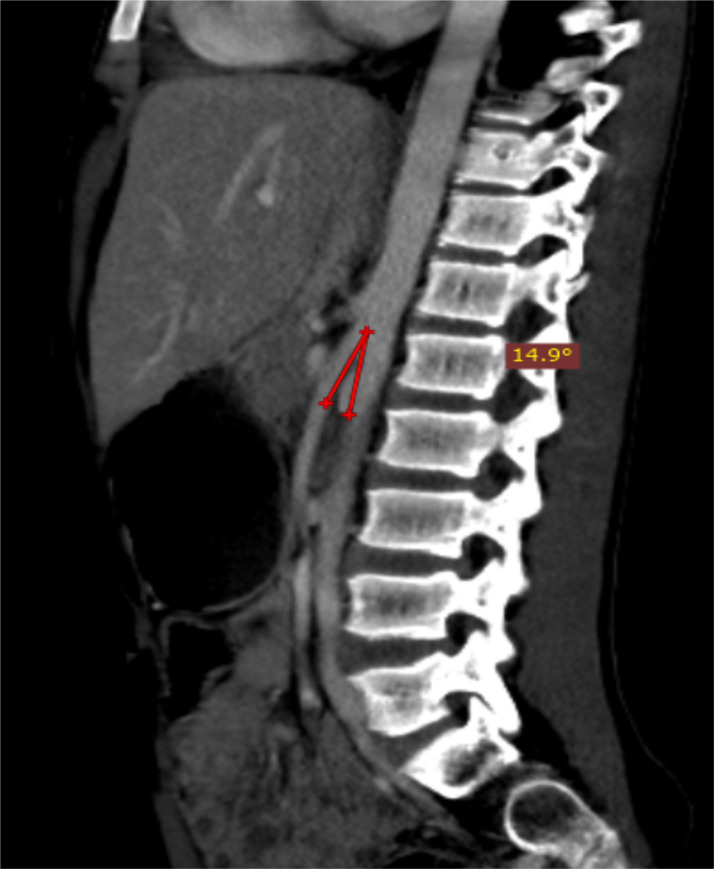
Abdominopelvic CT of a 12-year-old, known acute lymphocytic leukemia patient, on a post contrast mid sagittal image aortomesenteric angle (AMA) is 14.9^0^

Sagittal images were obtained for assessment of the branching configuration of the SMA from the aorta. AMD was measured as the maximum distance between the anterior margin of the aorta and the posterior aspect of the superior mesenteric artery at a level where the duodenum was crossing. For the angle measurement (AMA), a line was drawn between the root of SMA and an imaginary point on the SMA where SMA begins to descend parallel to the abdominal aorta. Measurements were obtained by electronic calipers. The angles were obtained by manual tracing and the degrees were automatically calculated. AMA < 22 degree and AMD of < 8 mm was used for definition of compression in this research(11, 12) (Fig. 2).

**Figure 3:**
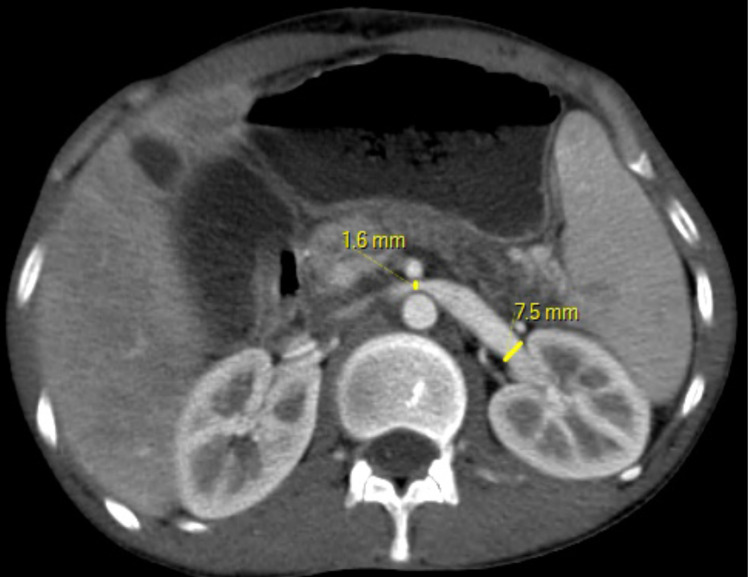
Abdominopelvic CT of a 30-year-old male patient presented with left chest pain and for work up for unknown primary. Post contrast axial abdominal CT at the level of renal vein shows compression of left renal vein as it passes between abdominal aorta and superior mesenteric artery (SMA).

The left renal vein diameter was measured on an axial image closest to the centerline of the vein as it crosses between the aorta and the SMA. On this same axial image, the anteroposterior distance between the aorta and the SMA was measured. Between this axis and the left kidney, the maximal axial diameter of the left renal vein was then measured. The compression ratio was calculated. In this study CR of greater than 3 and those with 2.7–3 and beak angle of greater than 32 degrees were included (14) (Fig. 3). Collateral veins of the LRV were deemed present when dilated enhanced left gonadal, lumbar, or adrenal veins were observed with a normal unenhanced right gonadal vein on arterial phase CT images.

**Statistical analysis**: Data were analyzed using statistical methods with SPSS version 25 software package. The average values of CT parameters are expressed as means ± standard deviations (SD). Categorical variables were reported using frequencies.

**Ethical consideration**: Ethical approval to conduct the study was obtained from ethics review committee of the department of radiology before the commencement of the study.

## Results

Out of 623 MDCT performed, a total of 513 (N = 513) patients were included in this study. Females comprised 277 (54 %) and 236(46%) were male. Mean age was 38 ± 20 (mean ± SD). Thirty-five (6.8%) had imaging features of SMA compression. Thirty-four (6.6%) of the patient fulfilled imaging criteria of nutcracker phenomenon and twenty-two (4.3%) of the patients showed celiac compression by MAL.

**Celiac axis compression:** The age range was 11–71years with mean of 47.41 ± 17.67 years. Males were 13 (59.1%) and females were nine (40.9%). In 19 out of 22 patients with celiac axis compression, the origin of the celiac artery was high (above the first lumbar vertebra). In 18 cases, MAL was located below the level of L1, indicating that a low insertion of MAL and normal in four patients (but low in relation to celiac origin in all 22 patients). Only three CT showed post stenotic dilatation of the celiac artery. No significant collaterals were demonstrated.

**Compression of the duodenum by Superior mesenteric artery (SMA)**: The imaging sign of SMA causing duodenal compression was observed in 27 females and 10 males. The age range was 12–79 years. Mean of AMA and AMD were 16.2 ± 2.96 degrees and 5.32 ± 1.18mm respectively. The degree of compression by AMA, in three (8.1%) of them was less than 12 degrees, 16 (43.2%) measured 18–22^0^, 18 (48.7%) measured 12–18^0^. Twenty-four (64.9%) of the SMA compressions had AMD of 4–6mm (table 1).

**Table 1:** Characteristics of aortomesenteric angle and aortomesenteric distance of the 37 study subjects with incidental findings of duodenal compression by superior mesenteric artery

Characteristics	Frequency (%)
Aortomesenteric angle (degree)	
<12	3 (8.1)
12–15	10 (27.1)
15–18	8 (21.6)
18–22	16 (43.2)
Aortomesenteric distance	
< 4mm	6 (16.2)
4–6mm	24(64.9)
6–8mm	7(18.9)

**Left renal vein compression by SMA (Nutcracker phenomenon)**: Out of 34 patients who have CT signs of nutcracker phenomenon, nine (26.5%) were males and 25(73.5%) were females. Mean age was 39.26 ± 16.93. The average AMA, AMD, Compression ratio and beak angle were 16.2 ± 3 degrees, 5.6 ± 1.2 mm, 4.2 ± 0.82 and 42.9 ± 9.79 degrees respectively.

## Discussion

The study was performed in a chronic care setting and a significant number of abdominopelvic MDCT were performed in a few months periods. The capability of sagittal reconstructions has improved visualization of the vasculatures, the adjacent structures and performance of measurements.

In our study the magnitude of celiac artery compression by median arcuate ligament, as defined by 50% luminal narrowing and hooked appearance on sagittal image with or without presence of ancillary findings was 4.3%. In most cases high origins of celiac artery and low insertion of MAL are risk factors for celiac compression. Our findings are in keeping with previous studies where they reported the incidence of celiac axis stenosis in asymptomatic individuals of 4–24%(16). In other study of with 5.1% incidence, all the eight subjects with celiac axis compression, the origin of the celiac artery was above the first lumbar vertebra. In six of the cases, MAL was located below the level of L1, indicating that a low insertion of MAL was responsible for the compression. Only three angiograms showed post stenotic dilatation of the celiac artery, and none showed significant collaterals(17).

SMA syndrome occurs relatively infrequently, its exact prevalence in the general population is extremely difficult to measure, although it has been estimated to be 0.1%–0.3% on the basis of gastrointestinal barium studies (12). However, in patients with scoliosis who undergo corrective spinal surgery, a prevalence of up to 2.4% has been reported (18). To the authors knowledge there are no studies reporting prevalence of compression of the duodenum by SMA in asymptomatic individuals. In our study, CT signs of duodenal compression by SMA was observed in 35 (6.8%) patients. The relative increment of the number can be justified by the fact that the study was performed in a chronic care setting, where a significant cases of cancers and other chronic illnesses are treated. These are conditions known to cause severe weight loss with resulting in a loss of retroperitoneal fat. The overall predominant lean body habitus in our population might have contributed for this number as well.

The occurrence of nutcracker phenomena in this study is 6.6 %, which is lower than previously reported (27.3%) (19) and a female preponderance (73.5%) which is not in line with the mentioned study.

This study has its own limitation, such as, the fact that it is a retrospective study, exploration of the clinical symptoms and follow up of patients was not possible. Ancillary findings are not included since it was difficult to assess small vessels in most of the patients and many of the images were not CT angiography. Additionally, CT does not assess the effect of respiration (inspiration vs expiration) which affects median arcuate ligament compression of celiac artery.

In conclusion, incidentally detected intraabdominal vascular compressions is common to asymptomatic patients. This result emphasizes that, vascular compression syndromes diagnosis should not be made on imaging alone.

## References

[1] Lamba R, Tanner DT, Sekhon S, McGahan JP, Corwin MT, Lall CG (2014). Multidetector CT of Vascular Compression Syndromes in the Abdomen and Pelvis. RadioGraphics.

[2] Eliahou R, Sosna J, Bloom AI (2012). Between a rock and a hard place: clinical and imaging features of vascular compression syndromes. Radiographics.

[3] Erden A, Yurdakul M, Cumhur T (1999). Marked increase in flow velocities during deep expiration: A duplex Doppler sign of celiac artery compression syndrome. Cardiovascular and interventional radiology.

[4] Welsch T, Büchler MW, Kienle P (2007). Recalling superior mesenteric artery syndrome. Digestive surgery.

[5] Srisajjakul S, Prapaisilp P, Bangchokdee S (2017). Imaging features of vascular compression in abdomen: Fantasy, phenomenon, or true syndrome. The Indian journal of radiology & imaging.

[6] Srisajjakul S, Prapaisilp P, Bangchokdee S (2017). Imaging features of vascular compression in abdomen: Fantasy, phenomenon, or true syndrome. The Indian journal of radiology & imaging.

[7] Harjola PT (1963). A RARE OBSTRUCTION OF THE COELIAC ARTERY. REPORT OF A CASE. Annales chirurgiae et gynaecologiae Fenniae.

[8] Brandt LJ, Boley SJ (1978). Celiac axis compression syndrome. A critical review. The American journal of digestive diseases.

[9] Manghat NE, Mitchell G, Hay CS, Wells IP (2008). The median arcuate ligament syndrome revisited by CT angiography and the use of ECG gating--a single centre case series and literature review. The British journal of radiology.

[10] Hines JR, Gore RM, Ballantyne GH (1984). Superior mesenteric artery syndrome: Diagnostic criteria and therapeutic approaches. The American Journal of Surgery.

[11] Konen E, Amitai M, Apter S, Garniek A, Gayer G, Nass S (1998). CT angiography of superior mesenteric artery syndrome. AJR American journal of roentgenology.

[12] Agrawal GA, Johnson PT, Fishman EK (2007). Multidetector row CT of superior mesenteric artery syndrome. Journal of clinical gastroenterology.

[13] Shokeir AA, el-Diasty TA, Ghoneim MA (1994). The nutcracker syndrome: new methods of diagnosis and treatment. British journal of urology.

[14] Kim KW, Cho JY, Kim SH, Yoon JH, Kim DS, Chung JW (2011). Diagnostic value of computed tomographic findings of nutcracker syndrome: correlation with renal venography and renocaval pressure gradients. European journal of radiology.

[15] Levin DC, Baltaxe HA (1972). High incidence of celiac axis narrowing in asymptomatic individuals. The American journal of roentgenology, radium therapy, and nuclear medicine.

[16] Park CM, Chung JW, Kim HB, Shin SJ, Park JH (2001). Celiac axis stenosis: incidence and etiologies in asymptomatic individuals. Korean journal of radiology.

[17] Soman S, Sudhakar SV, Keshava SN (2010). Celiac axis compression by median arcuate ligament on computed tomography among asymptomatic persons. Indian J Gastroenterol.

[18] Tsirikos AI, Jeans LA (2005). Superior mesenteric artery syndrome in children and adolescents with spine deformities undergoing corrective surgery. Journal of spinal disorders & techniques.

[19] Grimm LJ, Engstrom BI, Nelson RC, Kim CY (2013). Incidental detection of nutcracker phenomenon on multidetector CT in an asymptomatic population: prevalence and associated findings. Journal of computer assisted tomography.

